# New approaches to reprocessing of oxide nuclear fuel

**DOI:** 10.1007/s10967-012-2260-6

**Published:** 2012-09-26

**Authors:** B. F. Myasoedov, Yu. M. Kulyako

**Affiliations:** Vernadsky Institute of Geochemistry and Analytical Chemistry, Russian Academy of Sciences, ul. Kosygina 19, Moscow, 119991 Russia

**Keywords:** Spent oxide nuclear fuel, Dissolution, Ferric nitrate solution, Fission products

## Abstract

Dissolution of UO_2_, U_3_O_8_, and solid solutions of actinides in UO_2_ in subacid aqueous solutions (pH 0.9–1.4) of Fe(III) nitrate was studied. Complete dissolution of the oxides is attained at a molar ratio of ferric nitrate to uranium of 1.6. During this process actinides pass into the solution in the form of U(VI), Np(V), Pu(III), and Am(III). In the solutions obtained U(VI) is stable both at room temperature and at elevated temperatures (60 °C), and at high U concentrations (up to 300 mg mL^−1^). Behavior of fission products corresponding to spent nuclear fuel of a WWER-1000 reactor in the process of dissolution the simulated spent nuclear fuel in ferric nitrate solutions was studied. Cs, Sr, Ba, Y, La, and Ce together with U pass quantitatively from the fuel into the solution, whereas Mo, Tc, and Ru remain in the resulting insoluble precipitate of basic Fe salt and do not pass into the solution. Nd, Zr, and Pd pass into the solution by approximately 50 %. The recovery of U or jointly U + Pu from the dissolution solution of the oxide nuclear fuel is performed by precipitation of their peroxides, which allows efficient separation of actinides from residues of fission products and iron.

## Introduction

The progress of atomic power engineering in Russia directed towards on closed nuclear fuel cycle, which makes it necessary to develop novel innovation environmentally safe and economically advantageous low-waste technologies for reprocessing of spent nuclear fuel (SNF) both from the operating reactors and from fast reactors operating on mixed uranium–plutonium oxide fuel. To solve this problem SNF dissolution can be performed in subacid aqueous solutions of ferric nitrate, in which the acidity after the fuel dissolution is close to 0.1 M and allows direct recovery of U and Pu by precipitation of their fluorides, carbonates, oxalates, or peroxides [1, 2]. In this feature, the suggested SNF reprocessing technology differs from the Purex process in which the fuel is dissolved in concentrated HNO_3_ and then U and Pu are extracted from the strongly acidic solution with tributyl phosphate solutions in organic solvents with the subsequent stripping with subacid aqueous solutions (~0.1 M). As a result, 7–12 tons of acidic aqueous and organic solutions are formed as the waste per a ton of the reprocessed SNF. These solutions require further reprocessing and disposal. Thus, high acidity of the solutions in the SNF reprocessing technology is its major drawback. In this work we show that the use of subacid ferric nitrate solutions allows application of strong nitric acid to be discarded, which results in reduction in the waste solution volume, and mitigate the environmental impact of the waste. After the dissolution of the oxide fuel, the recovery of U and Pu from the solution is performed by precipitation of their peroxides. The behavior of the large number of fission products (FPs) in the course of dissolution of simulated SNF in ferric nitrate solutions and the recovery of uranium from these solutions was studied.

## Experimental

The commercial samples of UO_2_, U_3_O_8_, pellets enriched with ^235^U and MOX fuel (95.4 % ^238^UO_2_ and 4.6 % ^239^PuO_2_) in both powdered and granulated forms were used. Solid solutions of NpO_2_ and AmO_2_ in UO_2_ were synthesized from U–Np and U–Am oxalate mixtures, prepared in advance, by calcining these mixtures in an atmosphere of Ar +20 % H_2_ at 850 °C for 8 h. The data on the contents of FPs in irradiated fuel of WWER-1000 reactor (UO_2_, initial enrichment 5.5 % ^235^U, burn-up fraction 80 MW day kg^−1^), given in [3] we used for preparation of simulated SNF (SSNF). The calculated amounts of the elements––simulators of FPs in the form of their salts, manly nitrates or chlorides, were introduced into 3 M nitric acid solution of uranium. The solution obtained was evaporated up to solid residue and then calcinated in an oven at 850 °C in Ar  + 10 % H_2_ atmosphere. The relative content of FPs in the SSNF sample obtained (counting on the sum of metals) were as follows (wt%): Cs 0.60, Sr 0.19, Ba 0.35, Y 0.10, La 0.28, Ce 0.59, Nd 0.84, Zr 0.80, Mo 0.79, Tc 0.18, Ru 0.56, Pd 0.40 (ΣFP 5.68 %), and U 94.32 %. In SSNF reprocessing, the concentrations of most mentioned FPs in the resulting solutions were determined by atomic emission spectroscopy. It was shown that the presence of 100- and 200-fold amounts of U and Fe did not noticeably affect the accuracy of FPs determination. Weighed portions of the powdery oxides or SSNF samples were introduced into polypropylene centrifuge test tubes containing aqueous ferric nitrate solutions [Fe(NO_3_)_3_·9H_2_O] with pH 0.9–1.4. It should be noted that such acidity in the solutions of Fe(III) nitrate is caused by hydrolysis of these salts. Naturally, the solutions contain partially hydrolyzed soluble species Fe(OH)(NO_3_)_2_. The test tubes were hermetically stoppered and placed into a stirring device. After definite time intervals, the stirring was stopped. The suspensions were centrifuged, and the aqueous phases (mother liquors) were analysed. For that, aliquots of the solutions were deposited onto the targets (polished stainless steel disks), dried and calcined for measuring their α-activity, using α-spectrometer Alpha Analyst (Canberra). The uranium contents after dissolving MOX fuel samples was determined by spectrophotometry, because reliable radiometric determination of ^238, 235, 234^U at ~5 wt% ^239^Pu content in the solutions is impossible. The Tc content was found by measuring its β-activity on a UMF-2000 radiometer. The behavior of Cs and Fe was monitored after spiking the solutions with ^137^Cs and ^59^Fe, using a γ-ray spectrometer with a semiconductor germanium detector (Canberra). The concentrations were calculated from the γ-activity using Genie-2000 program. The oxidation states of U, Pu, Np, and Am in solutions were determined from the electronic absorption spectra recorded with a Unicam UV-340 spectrophotometer. The pH was measured with a Mettler Toledo MP230 pH meter with a combined glass electrode (Hanna Instrument HI 1131B) calibrated using buffer pH standards (pH 1–13, Merck).

## Results and discussion

### Dissolution of uranium oxides in subacid ferric nitrate solutions

**Fig. 1:**
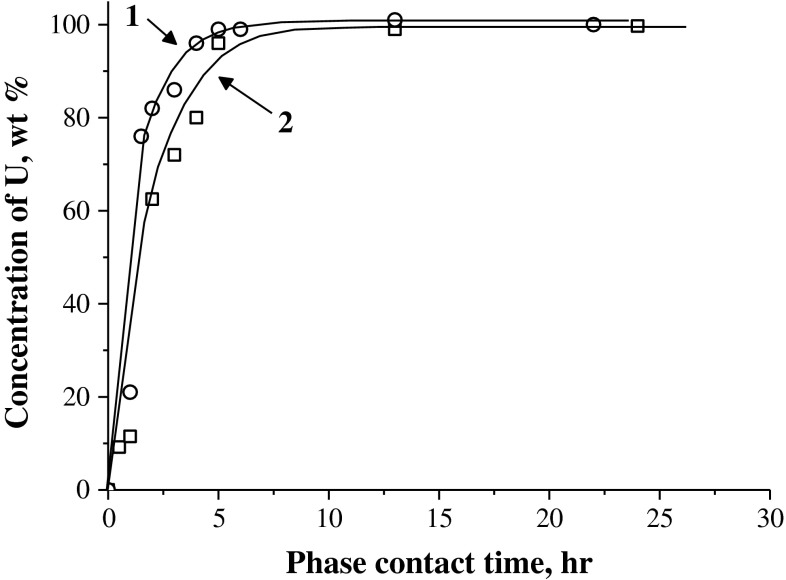
Kinetics of dissolution of UO_2_ (*1*) and SSNF (*2*) samples in ferric nitrate solutions (pH ~ 1; *t* ~ 22 °C; molar ratios Fe(NO_3_)_3_·9H_2_O:UO_2_/SSNF = 2)

The kinetics of dissolution of pure UO_2_ (Fig. 1, curve 1) and of the SSNF sample with FPs (Fig. 1, curve 2) under the same conditions are practically identical. Quantitative transfer of U from UO_2_ and SSNF samples into the solutions occurs within 5 h. As in the dissolution of UO_2_, the SSNF dissolution is accompanied by a decrease in the solution acidity (pH changes from 0.5 to ~1.5) and by the formation of a yellowish gray suspension of a basic salt, iron(III) dihydroxonitrate––Fe(OH)_2_(NO)_3_, which does not capture uranium in the course of precipitation from the solution. Uranium is not captured by the precipitate even at its high content in the solution (~300 g L^−1^) when the nitrate solution containing U(VI) is concentrated by evaporation at 60 °C (Table 1). Thus, the presence of precipitates of basic Fe(III) salts in solutions does not affect the uranium content. U(VI) solutions containing nitrate anions and Fe(III) and Fe(II) cations are stable for a long time both at elevated temperatures (60 °C) and at high uranium concentrations up to those commonly attained in nitric acid process solutions (6–8 M HNO_3_, 60–80 °C) and equal to ~300 g L^−1^.

**Table 1:** Content of U(VI) in the precipitate of basic ferric nitrate at 60 °C in relation to the U(VI) content in the solution

U(VI) in solution		U(VI) in precipitate^a^
Concentration (mg mL^−1^)	Weight (mg)	Weight (mg)
44.1	1,498 ± 25	0.18 (~0.01)
65.7	1,510 ± 30	0.15 (~0.01)
123.2	1,478 ± 19	0.20 (~0.01)
248.3	1,490 ± 20	0.13 (~0.01)

^a^The U(VI) percentage relative to its content in the solution given in parentheses

**Fig. 2:**
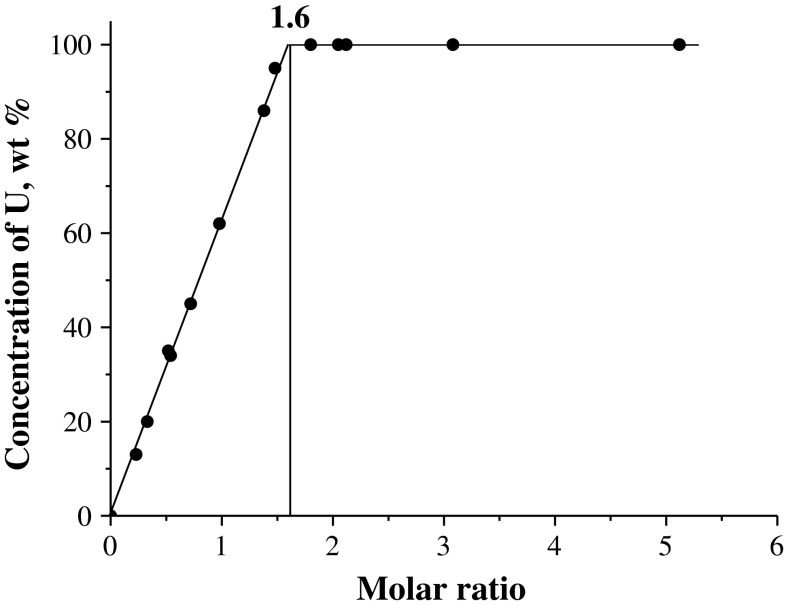
Degree of UO_2_ dissolution in the solutions of ferric nitrate (pH ~ 1) as a function of the molar ratio of the reagent to UO_2_ at ~22 °C

The degree of UO_2_ dissolution in solutions with pH ~ 1 is plotted in Fig. 2 against the molar ratio of ferric nitrate to UO_2_. The data obtained show that complete dissolution of UO_2_ is attained at the molar ratio of Fe(III) nitrate to UO_2_ equal to 1.6 (Fig. 2). Therefore, in the course of dissolution of UO_2_ in the ferric nitrate solution, U(IV) is oxidized to U(VI) not only by Fe(III) cations:

$$ {\text{UO}}_{2} + 2{\text{Fe}}\left( {{\text{NO}}_{3} } \right)_{3} = {\text{UO}}_{2} \left( {{\text{NO}}_{3} } \right)_{2} + 2{\text{Fe}}\left( {{\text{NO}}_{3} } \right)_{2} , $$

and probably by nitrate anions:

$$ {\text{UO}}_{ 2} + 4 {\text{HNO}}_{ 3} = {\text{UO}}_{ 2} \left( {{\text{NO}}_{ 3} } \right)_{ 2} + 2 {\text{NO}}_{ 2} + 2 {\text{H}}_{ 2} {\text{O}} . $$

**Fig. 3:**
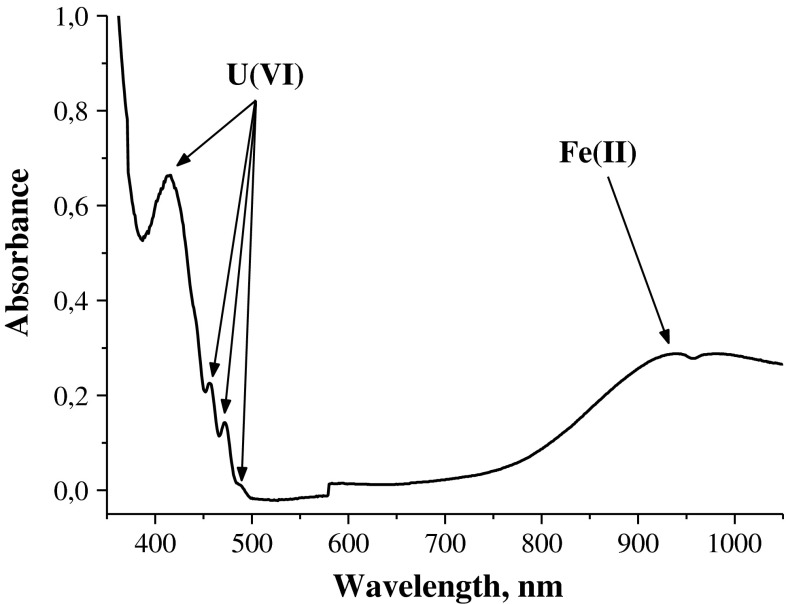
Spectrum of the solution after dissolution of UO_2_ in the ferric nitrate solution

Uranium exists in the solution in oxidation state 6+, which is confirmed by the presence of the U(VI) absorption bands at 420, 460, 470, and 490 nm in the solution spectrum (Fig. 3) after dissolution of UO_2_.

The broad absorption band at wavelengths from 700 to 1,050 nm with a maximum at 950 nm belongs to the Fe^2+^ cations arising in the course of the UO_2_ dissolution.

### Dissolution of MOX fuel and of solid solutions of NpO_2_ and AmO_2_ in UO_2_ in subacid ferric nitrate solutions

**Fig. 4:**
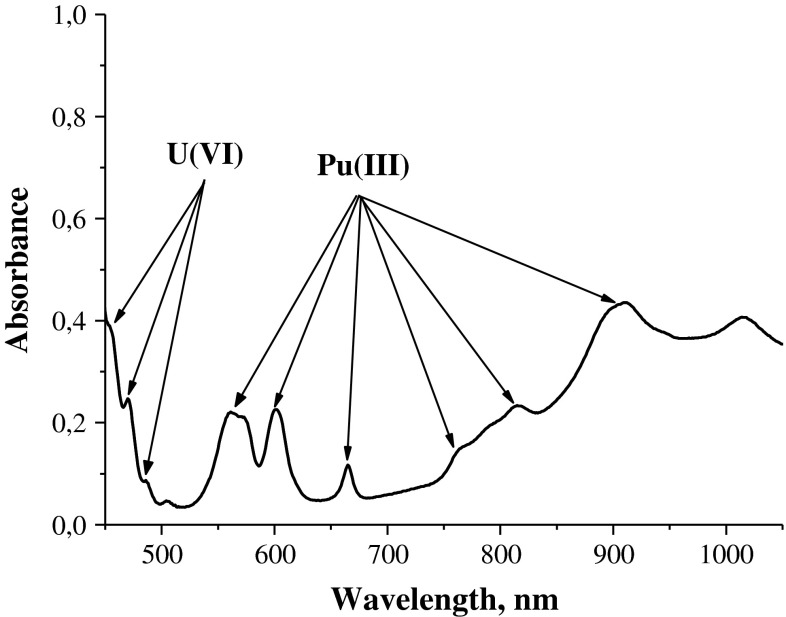
Spectrum of the solution after dissolution of MOX fuel in the ferric nitrate solution. (pH ~ 1), [Fe(NO_3_)_3_·9H_2_O] = 0.67 M, [U] = 0.212 M, [Pu(III)] = 5.1·10^−3^ M

Data on the dissolution of MOX fuel taken as a powder in a ferric nitrate solution (pH ~ 1) at ~22 °C are given in Table 2. Our results show that MOX fuel, like UO_2_, also dissolves in this medium. Figure 4 shows the spectrum of the solution obtained after dissolution of MOX fuel in the ferric nitrate solution.

**Table 2:** Dissolution of MOX fuel in a ferric nitrate solution (solution volume 4 mL, pH ~ 1, molar ratio Fe(NO_3_)_3_·9H_2_O:MOX fuel ~ 2)

MOX fuel taken (mg)			Found in solution (mg)	
Total	UO_2_	PuO_2_	UO_2_	PuO_2_
120.0 ± 0.1	114.5 ± 0.1	5.5 ± 0.1	111 ± 5	5.0 ± 0.5

As can be seen, U occurs in the solution in the form of U(VI), and Pu, in the form of Pu(III), because Pu(IV) initially present in the MOX fuel is reduced in the course of dissolution to Pu(III) with Fe(II) ions formed by oxidation of U(IV) to U(VI) with Fe(III). Solid solutions of mixed oxides NpO_2_–UO_2_ and AmO_2_–UO_2_ readily dissolve in the ferric nitrate solutions as well. The spectrophotometry analysis shows, that the dissolution of the mixed oxide UO_2_–NpO_2_ is accompanied by oxidation of Np(IV) to Np(V) with Fe(III) cations, similar to the oxidation of U(IV) to U(VI). On dissolution of UO_2_–AmO_2_, americium which is present in the crystal lattice of the mixed oxide as Am(IV) is reduced to Am(III) in the course of dissolution, as it is commonly observed on dissolution of AmO_2_ in mineral acids [4].

### Recovery of U and Pu by precipitation of peroxides from the ferric nitrate solution

The results of the experiments on the precipitation of U and Pu peroxides and their separation from Fe(III) are given in Table 3. As seen from the table, U and Pu are precipitated by 95 % from the solution and are virtually fully separated from Fe. The precipitate of the U and Pu peroxides after washing with water and drying can be calcined at 850 °C in a reducing atmosphere to the dioxides with the aim of the reuse. After the separation of actinide peroxides, it is possible to perform hydroxide precipitation of iron in the presence of ferrocyanide ions and to precipitate fission lanthanides, Tc, Sr, and Cs.

**Table 3:** Peroxide precipitation of U and Pu from nitrate solution (pH ~ 1) and their separation from the Fe(III)

Analyzed object	Element concentration (M)		
	U	Pu	Fe
Initial solution	6.8 × 10^−2^	4.4 × 10^−3^	0.15
Precipitate^a^	6.6 × 10^−2^	4.2 × 10^−3^	6.0 × 10^−5^
Mother liquor	0.2 × 10^−2^	0.2 × 10^−3^	~0.15

^a^The concentration of the elements was determined after dissolution of the mixed actinide peroxide precipitate in HNO_3_

### Behavior of fission products in dissolution of SSNF in the ferric nitrate solution

The results of determining the concentrations of FPs, U, and Fe in the solution and precipitate formed in the course of dissolution of SSNF samples in ferric nitrate solutions with pH ~ 1 are given in Table 4. As is seen, on dissolution of the fuel samples U quantitatively passes into the solution. In so doing, 60 wt% of Fe remains in the solution. A decrease in the Fe content in the solution is due to the precipitation of Fe(OH)_2_(An). Simultaneously with the dissolution of U, such FPs as Cs, Sr, Ba, Y, La, and Ce passes to the solution by more than 90 % of their content in the initial SSNF. Nd, Zr, and Pd were distributed between the solution and the forming precipitate of the basic Fe salt in approximately equal amounts. Mo, Tc, and Ru did not noticeably pass to the solution. This may be due to the fact that Mo, Tc, and Ru form in SSNF “light metal inclusions,” which, as known, are very sparingly soluble in concentrated HNO_3_. Thus, an advantage of using a subacid aqueous solutions of ferric nitrate for SSNF dissolution, compared to the Purex process, is the possibility of separation of U from Mo, Tc, and Ru (>95 %) and of partial separation from Nd, Zr, and Pd (~50 %) even at the stage of SSNF dissolution.

**Table 4:** Content of FPs, U, and Fe in the ferric nitrate solution (pH ~ 0.5) and in the precipitate formed on dissolution of SSNF

Element	Calculated concentration at complete SSNF dissolution (mg mL^−1^)	Found in solution		
		After dissolution of SSNF		After dissolution of precipitate of basic Fe salt (wt%)
		mg mL^−1^	wt%	
Cs	0.53	0.48	91	9
Sr	0.17	0.15	88	12
Ba	0.31	0.30	97	3
Y	0.09	0.08	89	11
La	0.25	0.24	96	4
Ce	0.52	0.50	96	4
Nd	0.74	0.32	43	57
Zr	0.71	0.26	37	63
Mo	0.70	0.005	0.7	99.3
Tc	0.16	0.01	6	94
Ru	0.49	0.001	0.2	99.8
Pd	0.35	0.14	40	60
U	84.0	83.5	99.4 ± 0.6	~0.01
Fe	42.0	25.2	60	40

### Behavior of fission products in the course of uranium recovery from subacid aqueous ferric nitrate solutions by precipitation of its peroxide

The results of experiments on studying the behavior of FPs in the course of U recovery by peroxide precipitation from solutions after SSNF dissolution are given in Table 5. As can be seen, the precipitation of uranium peroxide from the solution after the SSNF dissolution in ferric nitrate solutions ensured quantitative separation of U from practically all the FPs present in the solution (decontamination factor ~1,000) except Zr. In the step of the SSNF dissolution, it was distributed between the solution and the precipitate of the basic iron salt (Table 4), and in the step of the U recovery the dissolved Zr mostly co-precipitated with U (Table 5). Further decontamination of U and Pu from FPs (including Zr) can be performed using extraction refining operations with suitable extractants.

**Table 5:** Content of FPs, U, and Fe^a^ in the ferric nitrate solution with pH ~ 1 and in the precipitate formed on the SSNF dissolution

Element	Calculated concentration at complete dissolution of SSNF (mg mL^−1^)	Relative content of FPs (%)	
		After separation of U peroxide	After dissolution of U peroxide
Cs	0.48	~99.9	~0.1
Sr	0.15	~99.9	~0.1
Ba	0.30	~99.4	~0.6
Y	0.08	~99.9	~0.1
La	0.24	~99.9	~0.1
Ce	0.50	~99.6	~0.4
Nd	0.32	~99.5	~0.5
Zr	0.26	27	73
Mo	0.005	~100	<0.1
Tc	0.01	~100	<0.1
Ru	0.001	~100	<0.1
Pd	0.14	99.3	0.7
U	83.5	1.2	98.8
Fe	25.2	100	–

^a^The uncertainty in determination of the FPs content in the phases after U recovery did not exceed 10 %

## Conclusion

There was suggested an alternative method for SNF reprocessing using subacid solutions of ferric nitrate, which allow dissolving SNF and recovering actinides from these solution in the form of their peroxides and efficient separation of actinides from FPs and Fe. As compared to the currently used PUREX process, the number of steps of SNF reprocessing decreases, the liquid waste volume is considerably reduced, and the fuel reprocessing is made safer and more reliable.
